# Caregiver strain in progressive supranuclear palsy and corticobasal syndromes

**DOI:** 10.1007/s00702-021-02379-z

**Published:** 2021-07-22

**Authors:** Lukas Kellermair, Alexandra Fuchs, Christian Eggers, Petra Schwingenschuh, Mariella Kögl, Franz Fellner, Thomas Forstner, Stephanie Mangesius, Michael Guger, Gerhard Ransmayr

**Affiliations:** 1 Department of Neurology 2, Kepler University Hospital, Krankenhausstr. 9, 4021 Linz, Austria; 2 Department of Clinical and Health Psychology, Kepler University Hospital, Med Campus III, Linz, Austria; 3 Department of Neurology, Medical University of Graz, Graz, Austria; 4 Central Institute of Radiology, Kepler University Hospital, Linz, Austria; 5 Department of Applied Systems Research and Statistics, Johannes Kepler University, Linz, Austria; 6 Department of Neuroradiology, Medical University of Innsbruck, Innsbruck, Austria; 7 Faculty of Medicine, Johannes Kepler University, Linz, Austria

**Keywords:** Progressive supranuclear palsy, Corticobasal syndrome, Caregiver burden, Caregiver strain, Neuropsychiatric symptoms

## Abstract

Progressive supranuclear palsy (PSP) and corticobasal syndrome (CBS) progress relentlessly and lead to a need for care. Caregiving is often burdensome. Little is known about the course of caregiver burden (CB) in PSP and CBS patients. Longitudinal analysis of CB in family members caring for PSP and CBS patients. Single-center longitudinal pilot study in 68 newly diagnosed patients with probable PSP and CBS (52 Richardson’s syndrome; 1 progressive gait freezing of PSP; 15 CBS). Demographic, educational, occupational parameters, family status, motor functions (UPDRSIII, Hoehn and Yahr Score, Tinetti) and neuropsychological performance (CERAD Plus, Frontal Assessment Battery) were assessed, as well as behavioral and neuropsychiatric impairments (Frontal Behavioral Inventory, Neuropsychiatric Inventory), activities of daily living (ADL) and caregiver burden using the Caregiver Strain Index (CSI), in most patients also the Zarit Burden Interview (ZBI). Patients were followed up every 6 months for up to 2 years. Caregivers reported mild to moderate CB at baseline, which increased by 25–30% in 2 years and was significantly greater in PSP than in CBS. Risk for mental health problems increased over time, especially in female caregivers (depression). Important patient-related factors were apathy, aspontaneity, depression, irritability, disorganization, poor judgment, impairment of language, impairments in ADL, a high educational level of the patient and close family relationship. Behavioral symptoms and impaired ADL are the main patient-related factors of CB in PSP and CBS. CB can be severe and needs to be assessed repeatedly from the time of diagnosis to provide comprehensive support.

## Introduction

In 1964, J. Steele, J. Richardson and J. Olszewski described a disease characterized by vertical gaze and pseudobulbar palsy, postural instability, parkinsonism and mild dementia referred to as progressive supranuclear palsy (PSP), later as PSP-Richardson’s syndrome (Steele et al. 1964; Litvan et al. 1996a, b; Williams and Lees 2009). Four years later, a similar syndrome comprising asymmetric rigidity, akinesia, dystonia and apraxia was observed, from which the concepts of corticobasal degeneration and later corticobasal syndrome emerged (CBS; Ali and Josephs 2018; Rebeiz et al. 1968; Armstrong et al. 2013a). Motor, cognitive and also neuropsychiatric and behavioral symptoms, such as irritability, disinhibition, and depression, as well as neuropathological features overlap in PSP and CBS (Ali and Josephs 2018; Armstrong et al. 2013a; Kovacs 2015; Lamb et al. 2016; Gerstenecker et al. 2013). Language may be impaired and both disorders respond poorly to pharmacological therapies (Peterson et al. 2021; Armstrong et al. 2013a). Non-pharmacological supportive measures remain paramount and patients become in need of care (Williams and Lees 2009; Lamb et al. 2016). Caregivers of patients with chronically progressive disorders often report caregiver burdens (CB) resulting in psychological, somatic and financial distress and incompatibility of caregiving with occupation, family and private life (Uttl et al. 1998; Schmotz et al. 2017; Martinez-Martin et al. 2015). Neurocognitive and neuropsychiatric symptoms as well as age, sex, occupation, activities of daily living (ADL), connection to others and impaired health of the caregivers are potential factors of the quality of life of patients and CB in PSP and CBS (Wiblin et al. 2017; Bukki et al. 2016; Higginson et al. 2012; Knutson et al. 2008). Compared to Parkinson’s disease (PD) and Alzheimer’s disease (AD), little is known about the course of CB in PSP as compared to CBS patients (Uttl et al. 1998; Schmotz et al. 2017; Knutson et al. 2008; Schneider et al. 1999; Bleijlevens et al. 2015; Pillas et al. 2016). CB may vary between countries (Schneider et al. 1999; Bleijlevens et al. 2015) so that national studies make sense. Pilot studies could be an occasion for larger multi-center studies. Moreover, the knowledge of health professionals concerning CB in rare diseases may be limited (Wynn and Carpenter 2020).

Therefore, we aimed to do a longitudinal comprehensive analysis of patient- and caregiver-related factors of CB in newly diagnosed PSP and CBS patients consecutively referred to an Austrian primary care center for movement and cognitive disorders.

Our hypotheses were:

1. CB progresses over time in PSP and CBS.
2. CB might be more severe for PSP than for CBS because of early impairment of balance and gait.
3. Cognitive deterioration, impairment of behavior, motor impairments, dependence and neuropsychiatric symptoms contribute to CB in these disorders.

## Methods

### Diagnosis and recruitment

From 2009 to 2018, we recruited 52 consecutive patients with newly diagnosed probable PSP-Richardson syndrome (RS) and one patient with PSP-progressive gait freezing according to the NINDS-SPSP criteria (Litvan et al. 1996a), updated by Williams and Lees (Williams and Lees 2009), and 15 patients with probable CBS according to Litvan et al.1997, after 2013 according to Armstrong et al. (2013a,b). Patients and their caregivers who consented to the aims and procedures of regular longitudinal observation were included in a local frontotemporal lobar atrophy (FTLA) registry (together with AD, PD and frontotemporal dementia patients). During follow-up (FU) persons were repeatedly evaluated according to the most recent diagnostic criteria for PSP and CBS (Williams and Lees, 2009; Litvan et al. 1997; Armstrong et al. 2013a; Höglinger et al. 2017), and those primarily diagnosed according to Litvan et al. 1997 and Williams and Lees 2009 were retrospectively evaluated using these diagnostic criteria (Armstrong et al. 2013a; Höglinger et al. 2017).

### Study procedures

The study was approved by the local ethics review board (Ethikkommission des Landes Oberösterreich; Protocol Number 254). Written informed consent was obtained from the patients and the caregivers. All caregivers were family members of the patients. The baseline protocol comprised medical and family history, neurological and psychiatric symptoms and signs, routine blood laboratory and chemistry and educational, occupational and social parameters of the patients and the caregivers. Neuroimaging (cerebral MRI) was performed and MRI diagnostic characteristics analyzed as summarized below. Clinical examinations including ratings of motor symptoms, balance and depression were performed by movement disorder specialists (LK, GR, CE), while neuropsychological functions and neuropsychiatric symptoms were analyzed by a psychologist (AF) together with the caregivers. ADL were evaluated and CB was assessed by the caregivers using standardized scales and self-rating inventories (see below). All examinations were repeated every 6 months for up to 2 years (6, 12, 18 and 24 months after baseline; FU 1, 2, 3, 4) unless patients dropped out earlier because of severe disability, institutionalization, intercurrent disease, withdrawal of consent or other reasons.

### Clinical assessment scales

#### Caregiver burden

CB was assessed using the Caregiver Strain Index (CSI), a 13-item questionnaire for caregivers about presence/absence (score 1/ 0) of restrictions on work, leisure, family life, personal plans, impairment of sleep, emotional changes, shocking experiences, financial burden, exhaustion, and excessive demands of the client (Robinson 1983). CSI total scores of ≥ 7 indicate major caregiver strain.

During the course of the study the Zarit Burden Interview (ZBI) (Zarit et al. 1986) was added for better comparability of the findings of this study with the literature and the later initiated Austrian multi-center registry for AD patients (PRODEM; Knutson et al. 2008; Jabbari et al. 2020; Jones et al. 2017). The ZBI is a self-rating questionnaire comprising 22 questions about how caregivers experience restrictions on private life, contacts, social life and relationships, as well as financial burden, excessive demands of the client and health problems. Moreover, feelings of insufficiency and guilt, strong negative emotions, thoughts about giving up caregiving and overall CB are rated. The single item scores are based on a 5-point Likert scale ranging from 0 to 4 (0 = never, 4 = nearly always), with the total score ranging from zero (no) to 88 (maximum CB). Total scores of 0–20 indicate no or little, 21–40 mild to moderate, 41–60 moderate to severe, and > 60 severe CB. Total scores of 26 or higher indicate a significant risk for mental health (Schreiner et al. 2006).

CSI and ZBI address similar subject areas with few exceptions. The CSI asks for strain (according to the Oxford Dictionary, www.oxfordlearnersdictionaries.com: pressure, because of great demands) and the ZBI for burdens related to caregiving (Oxford Dictionary: duties, responsibilities, that cause difficulties). The ZBI is more detailed than the CSI and we had the impression that the wording of the CSI is more expressive. We hypothesized that, although there are differences between the two inventories, CSI and ZBI might lead to comparable results and the use of both inventories might, on the one hand, test the inner consistency of our findings and potentially corroborate the results. In this manuscript we use the term CB as an umbrella term for caregiver strain and CB.

At FU visits caregivers were also asked whether they had suffered from depressive episodes related to caregiving during the previous 6 months. Depression was defined as a period of anhedonia, sadness, exhaustion, apathy or anxiety resulting in treatment with antidepressants or anxiolytics.

#### Motor functions

The Unified Parkinson’s Disease Rating Scale, motor scale (UPDRS III) (Fahn and Elton 1987) and the Hoehn and Yahr scale (HYS) (Hoehn and Yahr 1967) were applied to quantify parkinsonian motor symptoms. We used the original UPDRS, version 3.0 (Fahn and Elton 1987), because it is relatively short and its scores would be comparable to those of other studies, for instance in PSP, which also used the original UPDRS (Uttl et al. 1998; Schmotz et al. 2017). Balance and gait were tested using the Tinetti Performance-Oriented Mobility Assessment. Total scores range from zero to 28. A score of 18 or less indicates a high risk for falls (Tinetti 1986).

#### Neuropsychological tests

For baseline evaluation, the CERAD plus neuropsychological battery was used (Schmid et al. 2014). The Mini-Mental State Examination (MMSE) was applied for global neuropsychological evaluation during FU (Folstein et al. 1975), the Frontal Assessment Battery (FAB) (Dubois et al. 2000) to assess frontal lobe functions at baseline and during FU. The FAB comprises five cognitive (similarities, lexical fluency, Luria motor series, conflicting instructions, Go-No-Go paradigm) and one neurological sub-test (grasping behavior). Total scores range from 18 (no evidence of frontal-executive deficits) to zero (maximum impairment).

#### The following neuropsychiatric and daily activities scales were used at baseline and during FU

Frontal Behavioral Inventory (FBI) (Kertesz et al. 1997): Twenty-four domains of neuropsychiatric and behavioral impairment are assessed for severity and frequency. Scores range from 0 (normal behavior) to 3 (severe impairment for most of the time), total scores from 0 to 72.

Neuropsychiatric Inventory (NPI) (Cummings 1997): Frequency (range 1–4 points) and severity (1–3 points) of ten categories of neuropsychiatric symptoms and two somatic functions (sleep and eating) are rated. Frequency and severity of the single symptoms are multiplied and summarized (total score 0 (symptomless), 144 (maximum impairment)). The symptom scores were clustered according to Garre-Olmo 2010 (Garre-Olmo et al. 2010) (psychotic, emotional, behavioral, somatic cluster).

Geriatric Depression Scale (GDS), short version (Yesavage et al. 1982): Patients are evaluated for the presence (score 1) or absence (score 0) of 15 depressive symptoms. A total score of 9 is the cut-off point for depression.

Lawton-Instrumental ADL (IADL) Scale (Lawton and Brody 1969): It comprises eight domains of IADL. The total scores range from 0 (severely impaired IADL and dependence) to 16 (no impairment).

Rivermead ADL Index (Whiting and Lincoln 1980): It contains 31 items about basic and more complex instrumental ADL, differentiating between self-sufficiency and two categories of household and outdoor activities. Single item scores range from 3 (independent) to 0 (failure), total scores from 93 (unimpaired ADL) to 0 (total dependence).

Barthel Index (BI) (Mahoney and Barthel 1965): The BI quantifies basic ADL functions. Total scores range from 0 (full dependence) to 100 (no impairment).

### Neuroimaging

Forty-nine PSP and all CBS patients underwent cranial MRI on 1.5 Tesla MRI Scanners (SIEMENS MAGNETOM Avanto syngo MR B19), four PSP patients underwent cranial CT for medical reasons.

We used the common radiological criteria for PSP and CBS (Quattrone et al. 2008; Oba et al. 2005; Boxer et al. 2006).

### Statistics

All data were tabulated in a descriptive way using appropriate summary statistics. Between-group differences in categorical variables were compared using Fisher’s exact test or exact *χ*^2^ test. Assumptions of normal distribution for continuous variables were tested with the Kolmogorov–Smirnov test with Lilliefors correction. Normally distributed continuous variables were compared using Student’s *T* test or Welch’s *T* test for variance heterogeneity (verification with Levene’s test). The exact Mann–Whitney *U* test was applied for non-normally distributed continuous variables or ordinal variables.

To assess potential univariate correlation between variables, PSP and CBS patients were merged (because of clinical and pathological similarities) and Spearman’s rank correlation coefficient or a rank biserial correlation coefficient (if one of the variables was dichotomic) applied.

Using selected univariate, statistically relevant variables multivariate risk models were estimated with general estimation techniques. GEE techniques were used to account for repeated measurements over time. Furthermore, for the individual measurements after 12 and 24 months, the most influential factors in terms of highest standardized beta were presented using a linear regression model.

A *p* value ≤ 0.05 was taken as the two-sided statistical significance level. The statistical computing software R, Version 3.6.1 (R Foundation for Statistical Computing, Vienna, Austria), was used.

## Results

### Patients: Demographic and clinical parameters at baseline (Table 1)

Baseline demographic and clinical data of the included patients are summarized in Table 1.

**Table 1:** Patients Demographic and clinical data at baseline

Variable	All patients (*n* = 68)	PSP (*n* = 53)	CBS (*n* = 15)	*p* values^§^
Age	71.9 ± 7.3 (75)	72.4 ± 6.8 (73)	70.3 ± 9.2 (71)	0.54
Female*	27 (39)	20 (37)	7 (46)	0.56
Male*	41 (61)	33(63)	8 (54)	0.56
Motor symptom duration (months)	34.9 ± 24.8 (29)	38.8 ± 25.1 (30)	22.0 ± 19.2 (12)	0.01
UPDRS III total score	37. ± 15.6 (36)	38.3 ± 15.8 (36.5)	32.6 ± 14.8 (30.5)	0.37
Hoehn and Yahr score	1.7 ± 0.9 (2)	1.8 ± 0.9 (2)	1.4 ± 0.9 (2)	0.15
Tinetti total score	21.1 ± 6.5 (23)	14.9 ± 7.6 (15)	21.8 ± 6 (24)	0.02
MMSE sum score	25.4 ± 4.2 (27)	25.1 ± 4.5 (27)	26.2 ± 3.2 (27.5)	0.43
FAB total score	12.7 ± 4.0 (13)	12.2 ± 4.2 (13)	14.4 ± 2.8 (15)	0.08
FBI total score	14.8 ± 13.1 (11)	17.0 ± 13.2 (13.5)	7.3 ± 9.4 (4)	< 0.001
NPI total score	12.8 ± 15.2 (7)	15.0 ± 16.0 (7)	4.7 ± 7.2 (2)	< 0.001
GDS score	3.4 ± 2.5 (3)	3.8 ± 2.5 (4)	2.0 ± 2.1 (1.5)	0.02
Barthel Index	77.6 ± 24.3 (85)	75.1 ± 23.2 (80)	86 ± 26.9 (100)	0.01
IADL total score	7.7 ± 4.5 (7)	6.9 ± 4.3 (7)	10.5 ± 4.1 (9.5)	< 0.001
Rivermead ADL total Score	55.9 ± 27.3 (62)	51.6 ± 27.4 (57)	69.43 ± 20.7 (73)	0.03

For each value with the exception of sex mean value ± standard deviation (median) is shown

*n (%)

^§^Comparison of PSP and CBS

*ADL* activities of daily living, *CBS* Corticobasal Syndrome, *GDS* Geriatric Depression Scale-15 (short form), *FAB* frontal assessment battery, *FBI* frontal behavioral inventory, *IADL* Lawton-Instrumental Activities of Daily Living Scale, *MMSE* Mini-Mental State Examination, *NPI* neuropsychiatric inventory, *PSP* progressive supranuclear palsy, *UPDRS III* Unified Parkinson’s Disease Rating Scale (III)

Mean age was 71.9 years, 61% of the study patients were male. Mean duration of motor symptoms was around 3 years for PSP and 2 years for CBS. Motor functions were on average moderately to markedly affected (UPDRS III, HYS, Tinetti total scores), and cognition (MMSE, FAB) mildly to moderately impaired in both groups. PSP patients exhibited mild to moderate, CBS patients less severe neuropsychiatric and behavioral impairments (FBI, NPI). Depression scores were low in both groups (GDS). ADL (BI, IADL and Rivermead ADL) were impaired in PSP and to a lesser degree in CBS patients.

### Comparison of PSP and CBS patients at baseline (Table 1)

Duration of motor symptoms was significantly shorter for CBS than for PSP. Tinetti, FBI, NPI and GDS total scores indicated less impairment of balance and milder neuropsychiatric and behavioral symptoms for CBS than for PSP. ADL were less impaired in CBS than in PSP patients (BI, IADL, Rivermead ADL). Age, male/female ratio, UPDRS III sum scores, HYS, MMSE and FAB total scores were similar in both groups.

### Patients: Education years, occupational and family status (Table 2)

Years of education and family and occupational status (except academic profession) were similar in PSP and CBS patients.

**Table 2:** Patients: Education, occupational and family status

Variable	All patients (*n* = 68)	PSP (*n* = 53)	CBS (*n* = 15)
Years of education*	11.0 ± 2.7 (11)	10.9 ± 2.9 (11)	11.2 ± 2.1 (11)
Occupational status^§^			
Mainly physical	22 (33)	19 (36)	3 (20)
Mainly not physical	32 (49)	22 (42)	10 (66)
Self-employed	5 (8)	4 (8)	1 (7)
Academic	4 (6)	3 (7)	1 (7)
Household	3 (4)	3 (7)	0 (0)
Family status^§^			
Married	47 (72)	36 (69)	11 (73)
Widowed	15 (23)	11 (22)	4 (27)
Divorced	2 (3)	2 (5)	0 (0)
Single	1 (1)	1 (2)	0 (0)
Partnership	1 (1)	1 (2)	0 (0)

*Mean ± standard deviation (median). ^§^*n* (%). Abbreviations see Table 1

### Caregiver-related parameters at baseline including caregiver burden (Table 3)

Caregivers were younger than patients; 72% (*n* = 49) were female. Age and years of education were similar for female and male caregivers. Caregivers were mainly partners or first-degree relatives (daughters or sons) of the patients. There were no statistical differences in occupational status or close family relationship (i.e., partner or first-degree relative) between female and male caregivers. A trend to a larger proportion of persons with academic qualification was observed among male caregivers. Most caregivers were retired (*n* = 51, 79%).

Completed CSI were available at baseline in 62, ZBI in 52 patients. CB was on average mild to moderate for PSP and low for CBS (CSI and ZBI total scores; for levels of significance, see Table 3). There was no statistical difference in CB between female and male caregivers. However, depressive episodes related to caregiving were more frequent in female than in male caregivers (49 vs. 23%; *p* = 0.04; OR 3.3; RR 2.2). Of 53 caregivers 44 (77%) lived in the same household with the patient.

**Table 3:** Caregivers: Demographic, occupational parameters at baseline, relationship to the patient

Variables	All caregivers (*n* = 68)^†^	Female caregivers (*n* = 49)	Male caregivers (*n* = 19)	*p* values*
Age	65.4 ± 10.7 (68)	67.7 ± 10.3 (70)	65.4 ± 10.7 (68)	0.77
Years of education	10.9 ± 2.7 (11)	10.9 ± 2.6 (11)	10.9 ± 2.7 (10)	0.32
ZBI	21.7 ± 13.6 (22)	22.3 ± 14.9 (23)	20.2 ± 10.1 (21.5)	0.68
CSI	3.5 ± 2.8 (3)	3.5 ± 2.9 (3)	3.5 ± 2.3 (4)	0.82
Occupational status				
Mainly physical	19 (28)	13 (27)	6 (34)	0.53
Not physical	32 (49)	25 (53)	7 (38)	0.30
Self-employed	3 (6)	2 (5)	1 (6)	0.82
Academic	7 (11)	3 (7)	4 (22)	0.06
Household	4 (6)	4 (8)	0 (0)	0.20
Relationship to the patient				
Partner	42 (65)	30 (63)	12 (66)	0.83
1st degree relative	16 (24)	12 (26)	4 (23)	0.78
Other	7 (11)	5 (11)	2 (11)	0.95
Retirement status				
Retired	51 (79)	36 (71)	15 (29)	0.84

	All caregivers	Caregivers of PSP patients	Caregivers of CBS patients	*p* values^§^
ZBI (*n* = 53)	21.7 ± 13.6 (22)	23.7 ± 12.5 (23)	13.1 ± 15.3 (10)	0.02
CSI (*n* = 62)	3.5 ± 2.8 (3)	4.1 ± 2.8 (4)	1.6 ± 1.6 (1)	0.01

^†^Occupational status and caregiver-patient relationship missing in 3 caregivers of PSP patients *Comparison of female and male caregivers and ^§^caregivers of PSP and CBS patients, *p* values

Age, years of education, ZCBI and CSI scores are indicated as means ± standard deviations (medians), occupational status and relationship to the patient as *n* (%); *CSI* Caregiver Strain Index, *ZBI* Zarit Burden Interview

CSI and ZBI correlated highly significantly (Spearman rank correlation, *p* < 0.0001).

### Longitudinal analysis of assessed parameters (Table 4)

Patients and caregivers dropped out during FU for reasons summarized in the Methods section.

**Table 4:** Patients: Clinical parameters of PSP and CBS patients and caregiver burden at baseline and at follow-up visits

Variables		Baseline (*n* = 68)	FU 6 months (*n* = 42)	FU 12 months (*n* = 35)	FU 18 months (*n* = 25)	FU 24 months (*n* = 17)
HYS	1.7 ± 0.9 (2)		1.8 ± 1.1 (2)	2.4 ± 1.0 (2)	2.4 ± 0.9 (3)	2.4 ± 0.6 (3)
UPDRS III	37.0 ± 15.6 (36)		45.4 ± 18.6 (45.5)	48.8 ± 17.1 (53.5)	49 ± 16.6 (51)	53.9 ± 16.9 (55)
Tinetti-Total	16.7 ± 7.8 (18)		13.9 ± 9.3 (15)	12.8 ± 9.1 (13)	11.8 ± 10.1 (10.5)	11.1 ± 10.5 (12)
Neuropsychological and neuropsychiatric scales						
FAB-Total	12.7 ± 4.1 (13)		13.3 ± 4.1 (14.5)	13.7 ± 3.8 (15)	12.9 ± 4.2 (14)	10.9 ± 4.9 (10)
MMSE	25.4 ± 4.2 (27)		26.0 ± 4.4 (25)	25.1 ± 4.4 (26)	25.3 ± 4.3 (26)	23.4 ± 4.2 (25)
GDS	3.4 ± 2.5 (3)		4.2 ± 2.7 (4)	4.5 ± 3.06 (4.5)	4.7 ± 3.87 (5)	3.6 ± 2.5 (4.5)
NPI	12.8 ± 15.2 (7)		18.6 ± 19.7 (13)	18.7 ± 17.75 (14)	20.1 ± 21 (14.5)	16.2 ± 19.3 (12)
FBI	14.9 ± 13.1 (11)		15.9 ± 13.2 (14)	17.6 ± 15.1 (12)	17.9 ± 13.1 (19)	15.5 ± 10.6 (12)
ADL-scales						
Barthel Index	77.6 ± 24.3 (85)		75.4 ± 24.5 (87.5	67.4 ± 24.9 (70)	62.3 ± 29.2 (60)	55.8 ± 29.3 (42.5)
IADL	7.7 ± 4.5 (7)		6.3 ± 4.5 (5)	5.3 ± 3.7 (4)	5.9 ± 4.9 (6)	3.9 ± 3.8 (2)
Rivermead ADL	55.9 ± 27.3 (62)		48.1 ± 26.6 (49.5)	41.1 ± 27.4 (41)	37.2 ± 31.3 (35)	30.7 ± 27.9 (24.5)
Caregiver burden						
CSI sum score	3.5 ± 2.8 (3)		4.4 ± 3.4 (5)	4.8 ± 3.2 (5)	5.1 ± 3.2 (5)	4.7 ± 2.8 (5)
ZBI sum score	21.7 ± 13.6 (22)		23.6 ± 16.1 (25)	26.7 ± 15.8 (28)	26.6 ± 14.9 (29)	29.2 ± 17.5 (31)
ZBI sum score ≥ 26	20 (37%)		18 (47%)	17 (53%)	13 (56%)	7 (53%)

*ADL* activity of daily living, *CBS* Corticobasal Syndrome, *CSI* Caregiver Strain Index, *GDS* Geriatric Depression Scale-15 (short form), *FAB* frontal assessment battery, *FBI* frontal behavioral inventory, *FU* follow-up, *HYS* Hoehn and Yahr-Scale, *IADL* Lawton and Brody-Instrumental Activities of Daily Living, *MMSE* Mini-Mental State Examination, *NPI* neuropsychiatric inventory, *PSP* progressive supranuclear palsy, *UPDRS III* Unified Parkinson’s Disease Rating Scale (III), *ZBI* Zarit Burden Interview

CSI total scores at baseline were comparable in female and male patients (CSI 3.5 versus 3.5).

HYS, UPDRS III, Tinetti total scores and ADL (BI, IADL and Rivermead ADL) deteriorated from baseline to FU4. Neuropsychological results (MMSE, FAB) were stable from baseline to FU3 (18 months) and deteriorated from FU3 to FU4 (24 months: FAB: 12.9–10.9 and MMSE: 25.3–23.4). Neuropsychiatric and behavioral symptoms (NPI, FBI, GDS) deteriorated from baseline to FU3 (NPI: 12.8–20.9; FBI: 14.9–17.9 and GDS: 3.4–4.7) and were less severe at FU4 in the seventeen patients remaining in the study until FU4 than at FU3. CSI sum scores for the entire collective of caregivers deteriorated from baseline to FU3 (3.5–5.1) and were slightly lower at FU4 than at FU3. ZBI sum scores progressed from baseline to FU4 (21.7–29.2).

CSI sum scores for caregivers of PSP patients increased from mean 4.1 ± 2.8 (SD) at baseline to 5.2 ± 3.2 at FU4, ZBI sum scores from 23.7 ± 12.5 to 32.3 ± 18.1. In caregivers of CBS patients the CSI sum scores were 1.6 ± 1.6 at baseline and 3.3 ± 4.3 at FU4. The respective ZBI scores were 13.9 ± 17.6 and 18.8 ± 20.3.

The proportion of caregivers with a CSI total score ≥ 7 (major caregiver strain; Robinson 1983) was 16% at baseline and increased to 30% until FU4. The most frequent complaints were “Changes in personal plans” (e.g., I had to turn down a job; could not go on vacation; 14.8%), “Caregiving is confining” (e.g., helping restricts free time; cannot go visiting; 13.3%) and “There have been family adjustments” (e.g., because helping has disrupted routine; there has been no private life; 11.8%).

### Caregiver burden and caregiver strain: correlations with motor, neuropsychiatric and neuropsychological parameters and activities of daily living

At baseline, FU1 and FU2 CSI total scores correlated significantly with HYS (range of *p* values < 0.001–0.02) and Tinetti total scores (*p* = 0.01–0.04). Moreover, CSI correlated with UPDRS III total scores at baseline and FU1 (*p* = 0.01 and 0.009). At baseline, FU1 and FU2 CSI total scores correlated with the BI (range of p values from < 0.001 to 0.002) and the IADL total scores (*p* < 0.001–0.03). ZCBI also correlated significantly with the BI (*p* < 0.0001–0.005) and IADL total scores (*p* < 0.001–0.004) at baseline, FU1 and FU2 as well as at FU4 (*p* = 0.05).

CSI total scores correlated significantly with neuropsychiatric impairments and behavioral and personality changes (NPI total scores, from baseline to FU4: range of *p* values < 0.001–0.007, FBI total scores from baseline to FU4: *p* < 0.001–0.004). Similar correlations were found for the ZBI total scores. The ZCBI correlated with the NPI total scores from baseline to FU3 (*p* < 0.001–0.003) and with the FBI total scores from baseline to FU4 (*p* < 0.001–0.003).

Highly significant correlations were found between baseline CSI total scores and the NPI behavioral (sum of elation/euphoria, apathy, disinhibition and aberrant motor behavior scores) and emotional clusters (agitation/irritability, depression/dysphoria, anxiety and irritability; *p* < 0.0001). A less significant correlation was found with the somatic (sum of eating and sleeping behavior scores, *p* = 0.04) and no correlation with the psychotic cluster (sum score of delusions, hallucinations, *p* > 0.05).

CSI total scores correlated with GDS scores at baseline and FU1 (*p* ≤ 0.001 and 0.01) and with FAB (*p* = 0.002 and 0.003, respectively) and MMSE sum scores at baseline and FU1. (*p* = 0.03–0.04).

ZBI total scores showed similar correlations with GDS at baseline and FU1 (*p* < 0.001 to 0.01) and with FAB (*p* = 0.005–0.007) at baseline and FU1, but did not correlate with MMSE sum scores at any point.

### Indicators of risk for caregiver burden (Table 5)

GEE techniques were used to identify risk factors for CB over time. Included in this analysis were all parameters discussed so far. Risk factors for CB (higher CSI total scores) were impaired ADL (Rivermead ADL; *p* < 0.001) and frontal lobe-type behavioral abnormalities (FBI total score; *p* < 0.001). Moreover, a higher educational level of the patient was an influential risk factor for higher ZCBI (*p* = 0.01), but not for higher CSI (*p* > 0.05).

Since the FBI total score was the most influential risk factor for CB, a detailed regression analysis of the FBI components and CSI and ZBI sum scores was performed (significant results shown in Tables 5, 6).

**Table 5:** Risk model of CSI

Most influential risk factors at BL and after 12 and 24 months. Regression analyses				
Parameter	Standardized coefficients	Standard-deviation	Adjusted *R*^2^	*p* value
FBI- BL				
FBI-Apathy	0.319	0.309	0.357	0.01
FBI-Irritability	0.243	0.362	0.357	0.03
FBI-Logopenia	0.262	0.407	0.357	0.02
FBI after 12 months				
FBI-Aphasia and verbal apraxia	0.413	0.374	0.791	0.005
FBI-Inattention	0.356	0.334	0.791	0.003
FBI after 24 months				
FBI-Aphasia and verbal apraxia	1.259	0.462	0.832	< 0.001
FBI-Logopenia	0.648	0.480	0.832	0.01

*CSI* Caregiver Strain

Risk factors for developing CSI over time (overall time effect, *p* = 0.297) at BL, 12 months and 24 months

Variables analyzed in the multivariate risk models: Age, Barthel Index, Body Mass Index, duration of motor symptoms, education years, family relationship, FBI Frontal Behavioral Inventory; Frontal Assessment Battery, Neuropsychiatric Inventory, occupational status, Rivermead ADL, sex of patient, sex and age of caregiver, Unified Parkinson’s Disease Rating Scale III

**Table 6:** Risk model of ZBI

Most influential risk factors at BL and after 12 and 24 months. Regression analyses				
Parameter	Standardized coefficients	Standard-deviation	Adjusted *R*^2^	*p* value
FBI- BL				
FBI-Apathy	0.299	1.472	0.507	0.01
FBI-Aspontaneity	0.237	1.972	0.507	0.02
FBI-Concreteness	0.480	2.062	0.507	< 0.001
FBI after 12 months				
FBI-Apathy	0.416	2.758	0.634	0.02
FBI-Inattention	0.503	2.502	0.634	0.01
FBI after 24 months				
FBI-Aphasia and verbal apraxia	0.687	3.354	0.530	< 0.001
FBI-Poor judgment	0.652	3.586	0.530	0.001

*ZBI* Zarit Caregiver Burden Interview

Risk factors for developing ZBI over time (overall time effect, *p* = 0.297) at BL, 12 months and 24 months

Variables analyzed in the multivariate risk models: Age, Barthel Index, Body Mass Index, duration of motor symptoms, education years, family relationship, FBI; Frontal Behavioral Inventory; Frontal Assessment Battery, Neuropsychiatric Inventory, occupational status, Rivermead ADL, sex of patient, sex and age of caregiver, Unified Parkinson’s Disease Rating Scale III

## Discussion

Little is known about the course of the burden of care for patients with PSP and CBS, in particular during the first years after diagnosis (Uttl et al. 1998; Schmotz et al. 2017; Bukki et al. 2016; Knutson et al. 2008; Pillas et al. 2016; Armstrong et al. 2013b; Jabbari et al. 2020). Our patients were consecutively recruited and carefully diagnosed using established clinical and neuroimaging criteria (Höglinger et al. 2017; Armstrong et al. 2013a; Quattrone et al. 2008). Because of clinical and presumed neuropathological overlaps between PSP and CBI in our collective and in agreement with the literature, PSP and CBS patients were merged for an analysis of factors underlying caregiver burden (Bukki et al. 2016; Armstrong et al. 2013b; Jabbari et al. 2020).

Our study has limitations.

First, at baseline 68 patient and caregiver diads were seen. However, mainly due to the quick progression of PSP and CBS numerous patients were lost to FU. A substantial loss to FU, however, is typical for these disorders (Higginson et al. 2021).

Second, one could argue that the lack of neuropathological diagnosis limits the validity of the data. One could also criticize the lack of CSF analyses for etiological diagnoses. Our study comprises patients with clinically probable diagnoses, thus syndromes, and neuropathological confirmation or specification of the diagnosis would not be of added value for the aims of the study. Our cohort is demographically and clinically similar to larger collectives of CBS and PSP patients, so that we believe our data are valid, at least for patients in the initial phase of the disorders (Jabbari et al. 2020).

Third, patients included in the study until FU3 and FU4 might have had slower disease progression than did participants who dropped out earlier. CB and CS might progress more rapidly in real life than shown in our study. On the other hand, the apparent partial stabilization of CB towards the end of FU might be due to an attenuation of behavioral and neuropsychiatric symptoms and a decrease in burdensome risky ambulation, but also accommodation of the caregivers to the challenges of caregiving.

Fourth, we did not assess quality of life (QoL) of the patients. However, QoL is a surrogate parameter for several clinical factors, such as severity of motor and neuropsychiatric symptoms (including depression), dementia, dependence and ADL, which were assessed in our study (Table 1). Moreover, patients with frontal lobe-type neurocognitive disorders including impairment of insight and empathy tend to fail to rate their QoL properly.

Our study demonstrates that CB is substantial in both disorders and increases during disease duration so that one-third of the caregivers of PSP patients were at risk for mental health disorders. We found that CB was more severe for PSP than for CBS at baseline and progressed with disease duration due to more severe and progressive neuropsychiatric symptoms and especially behavioral impairments, such as loss of empathy, aspontaneity, inattention, apathy, irritability, emotional flatness, apraxia of speech, impaired judgment and progressive communicative disabilities.

In the longitudinal analysis CB (CSI and ZBI total scores) correlated with motor impairment at baseline, but not later when motor symptoms appeared to play a lesser role than behavioral abnormalities or neuropsychiatric symptoms. These results correspond to the data published by Schmotz et al., who was not able to predict CB from motor symptoms in PD and PSP patients (Schmotz et al. 2017). CB also correlated with a wide spectrum of neuropsychiatric symptoms/clusters assessed by means of the NPI, except for psychotic symptoms, which were rare in theses diagnoses.

All caregivers were family members, mainly women, which corresponds to the literature (Martinez-Martin et al. 2015; Schneider et al. 1999; Bleijlevens et al. 2015). In agreement with the literature, CB was comparable in female and male caregivers (Knutson et al. 2008; Armstrong et al. 2013b; Southi et al. 2019). However, female caregivers more frequently reported depression, a new finding made in this study. The reasons are not clear and need to be investigated in future studies. Another novelty of our study is the knowledge gained about the care of PSP and CBS patients during initial phases of the disease. Interestingly, we did not find significant correlations between burden of care and patient age, caregiver age or caregiver occupational status, which is in contradiction to other reports in the literature (Tsai et al. 2021; Schneider et al. 1999; Iavarone et al. 2014). The correlations between educational level of the patient and close patient-caregiver relationship (partner or first-degree relative) with CB also need to be verified in future studies.

Compared to the literature on late-stage PSP (Schmotz et al. 2017; Kalampokini et al. 2020), caregivers in our study reported similar or less severe CB. In agreement with our findings the CLASP study also reported neuropsychiatric and non-motor features to be the leading factors for caregiver burden (Kalampokini et al. 2020). We were not able to find any data in the literature about CB in early stages of PSP. In this regard our study contributes to the knowledge about CB in the PSP-RS syndrome.

Compared to patients with early PD (Martinez-Martin et al. 2015; Jones et al. 2017), CB was similar or more severe in PSP and CBS patients. The proportion of caregivers reporting moderate to severe or severe CB (ZCBI sum score > / = 41, 53%) was similar to the findings for late-stage PD (48%) (Jones et al. 2017). In our series, CBS was seen to cause less CB than reported in the literature (Knutson et al. 2008; Armstrong et al. 2013b; Jones et al. 2017). In the literature several authors reported greater CB in patients with frontotemporal dementia, frontal behavioral variant (bvFTD) than in patients with CBS (Jones et al. 2017; Knutson et al. 2008; Armstrong et al. 2013b; Tessitore et al. 2018; Galvin et al. 2017), which they explained as earlier onset of significant neuropsychiatric and behavioral symptoms in bvFTD than in CBS (Knutson et al. 2008; Galvin et al. 2017; Tessitore et al. 2018; Jones et al. 2017). Accordingly, we found that the CB in CBS patients rose in parallel with the occurrence of behavioral disturbances in addition to motor dysfunction (Jones et al. 2017; Knutson et al. 2008; Galvin et al. 2017; Tessitore et al. 2018). When comparing our study and another neurodegenerative disease, namely AD, CB was seen to be more severe in PSP-RS and less severe in CBS than in recent studies on caregiving in AD (Ransmayr et al. 2018; Gronning et al. 2013). CB also has a stronger negative impact on health in PSP than in AD, CBS or PD.

In conclusion, the burden of caring for a patient with a neurodegenerative disease seems to be greater when the disease leads to the loss of the individual premorbid personality as opposed to diseases where motor and ADL dysfunctions are salient features. CB seems to reflect the overall severity of PSP and CBS, since it comprises most disease characteristics. Our results may contribute to a better understanding of the needs of family members caring for PSP and CBS patients. The present study may also contribute to improved comprehensive assessment of CB and CS in patients with PSP and CBS in everyday routine and in further studies.

Caregivers reported feelings of being left alone, fear of disease progression and of the future, restrictions on personal plans, extra costs, confinement, family adjustments, emotional irritations and health problems. Close monitoring of caregivers of PSP and CBS patients from the beginning of the disease and the provision of comprehensive support could stabilize CB and reduce its economic burden.

## Data Availability

The datasets generated and/or analyzed during the current study are available from the first and corresponding author on reasonable request.

## References

[CR1] Ali F, Josephs KA (2018). Corticobasal degeneration: key emerging issues. J Neurol.

[CR2] Armstrong MJ, Litvan I, Lang AE, Bak TH, Bhatia KP, Borroni B, Boxer AL, Dickson DW, Grossman M, Hallett M, Josephs KA, Kertesz A, Lee SE, Miller BL, Reich SG, Riley DE, Tolosa E, Troster AI, Vidailhet M, Weiner WJ (2013). Criteria for the diagnosis of corticobasal degeneration. Neurology.

[CR3] Armstrong N, Schupf N, Grafman J, Huey ED (2013). Caregiver burden in frontotemporal degeneration and corticobasal syndrome. Dement Geriatr Cogn Disord.

[CR4] Bleijlevens MH, Stolt M, Stephan A, Zabalegui A, Saks K, Sutcliffe C, Lethin C, Soto ME, Zwakhalen SM, RightTimePlaceCare C (2015). Changes in caregiver burden and health-related quality of life of informal caregivers of older people with Dementia: evidence from the European RightTimePlaceCare prospective cohort study. J Adv Nurs.

[CR5] Boxer AL, Geschwind MD, Belfor N, Gorno-Tempini ML, Schauer GF, Miller BL, Weiner MW, Rosen HJ (2006). Patterns of brain atrophy that differentiate corticobasal degeneration syndrome from progressive supranuclear palsy. Arch Neurol.

[CR6] Bukki J, Nubling G, Lorenzl S (2016). Managing advanced progressive supranuclear palsy and corticobasal degeneration in a palliative care unit: admission triggers and outcomes. Am J Hosp Palliat Care.

[CR7] Cummings JL (1997). The Neuropsychiatric Inventory: assessing psychopathology in dementia patients. Neurology.

[CR8] Dubois B, Slachevsky A, Litvan I, Pillon B (2000). The FAB: a Frontal Assessment Battery at bedside. Neurology.

[CR9] Fahn S, Elton RL, Fahn S, Marsden CD, Calne DB, Goldstein M (1987). UPDRS Development Committee. The Unified Parkinson’s Disease Rating Scale. Recent developments in Parkinson’s Disease.

[CR10] Folstein MF, Folstein SE, McHugh PR (1975). “Mini-mental state”. A practical method for grading the cognitive state of patients for the clinician. J Psychiatr Res.

[CR11] Galvin JE, Howard DH, Denny SS, Dickinson S, Tatton N (2017). The social and economic burden of frontotemporal degeneration. Neurology.

[CR12] Garre-Olmo J, Lopez-Pousa S, Vilalta-Franch J, de Gracia Blanco M, Vilarrasa AB (2010). Grouping and trajectories of neuropsychiatric symptoms in patients with Alzheimer’s disease. Part II: two-year patient trajectories. J Alzheimers Dis.

[CR13] Gerstenecker A, Duff K, Mast B, Litvan I, Group E-PS (2013). Behavioral abnormalities in progressive supranuclear palsy. Psychiatry Res.

[CR14] Gronning H, Kristiansen S, Dyre D, Rahmani A, Gyllenborg J, Hogh P (2013). Caregiver burden and psychosocial services in patients with early and late onset Alzheimer’s disease. Dan Med J.

[CR15] Higginson IJ, Gao W, Saleem TZ, Chaudhuri KR, Burman R, McCrone P, Leigh PN (2012). Symptoms and quality of life in late stage Parkinson syndromes: a longitudinal community study of predictive factors. PLoS ONE.

[CR16] Hoehn MM, Yahr MD (1967). Parkinsonism: onset, progression, and mortality. Neurology.

[CR17] Höglinger GU, Respondek G, Stamelou M, Kurz C, Josephs KA, Lang AE, Mollenhauer B, Muller U, Nilsson C, Whitwell JL, Arzberger T, Englund E, Gelpi E, Giese A, Irwin DJ, Meissner WG, Pantelyat A, Rajput A, van Swieten JC, Troakes C, Antonini A, Bhatia KP, Bordelon Y, Compta Y, Corvol JC, Colosimo C, Dickson DW, Dodel R, Ferguson L, Grossman M, Kassubek J, Krismer F, Levin J, Lorenzl S, Morris HR, Nestor P, Oertel WH, Poewe W, Rabinovici G, Rowe JB, Schellenberg GD, Seppi K, van Eimeren T, Wenning GK, Boxer AL, Golbe LI, Litvan I, Movement Disorder Society-endorsed PSPSG (2017). Clinical diagnosis of progressive supranuclear palsy: The movement disorder society criteria. Mov Disord.

[CR18] Iavarone A, Ziello AR, Pastore F, Fasanaro AM, Poderico C (2014). Caregiver burden and coping strategies in caregivers of patients with Alzheimer’s disease. Neuropsychiatr Dis Treat.

[CR19] Jabbari E, Holland N, Chelban V, Jones PS, Lamb R, Rawlinson C, Guo T, Costantini AA, Tan MMX, Heslegrave AJ, Roncaroli F, Klein JC, Ansorge O, Allinson KSJ, Jaunmuktane Z, Holton JL, Revesz T, Warner TT, Lees AJ, Zetterberg H, Russell LL, Bocchetta M, Rohrer JD, Williams NM, Grosset DG, Burn DJ, Pavese N, Gerhard A, Kobylecki C, Leigh PN, Church A, Hu MTM, Woodside J, Houlden H, Rowe JB, Morris HR (2020). Diagnosis across the spectrum of progressive supranuclear palsy and corticobasal syndrome. JAMA Neurol.

[CR20] Jones AJ, Kuijer RG, Livingston L, Myall D, Horne K, MacAskill M, Pitcher T, Barrett PT, Anderson TJ, Dalrymple-Alford JC (2017). Caregiver burden is increased in Parkinson’s disease with mild cognitive impairment (PD-MCI). Transl Neurodegener.

[CR21] Kalampokini S, Hommel A, Lorenzl S, Ferreira JJ, Meissner WG, Odin P, Bloem BR, Dodel R, Schrag AE (2020). Caregiver burden in late-stage parkinsonism and its associations. J Geriatr Psychiatry Neurol.

[CR22] Kertesz A, Davidson W, Fox H (1997). Frontal behavioral inventory: diagnostic criteria for frontal lobe dementia. Can J Neurol Sci.

[CR23] Knutson KM, Zamboni G, Tierney MC, Grafman J (2008). Neural correlates of caregiver burden in cortical basal syndrome and frontotemporal dementia. Dement Geriatr Cogn Disord.

[CR24] Kovacs GG (2015). Invited review: neuropathology of tauopathies: principles and practice. Neuropathol Appl Neurobiol.

[CR25] Lamb R, Rohrer JD, Lees AJ, Morris HR (2016). Progressive supranuclear palsy and corticobasal degeneration: pathophysiology and treatment options. Curr Treat Options Neurol.

[CR26] Lawton MP, Brody EM (1969). Assessment of older people: self-maintaining and instrumental activities of daily living. Gerontologist.

[CR27] Litvan I, Agid Y, Calne D, Campbell G, Dubois B, Duvoisin RC, Goetz CG, Golbe LI, Grafman J, Growdon JH, Hallett M, Jankovic J, Quinn NP, Tolosa E, Zee DS (1996). Clinical research criteria for the diagnosis of progressive supranuclear palsy (Steele-Richardson-Olszewski syndrome): report of the NINDS-SPSP international workshop. Neurology.

[CR28] Litvan I, Mega MS, Cummings JL, Fairbanks L (1996). Neuropsychiatric aspects of progressive supranuclear palsy. Neurology.

[CR29] Mahoney FI, Barthel DW (1965). Functional evaluation: the Barthel Index. Md State Med J.

[CR30] Martinez-Martin P, Rodriguez-Blazquez C, Forjaz MJ, Frades-Payo B, Aguera-Ortiz L, Weintraub D, Riesco A, Kurtis MM, Chaudhuri KR (2015). Neuropsychiatric symptoms and caregiver’s burden in Parkinson’s disease. Parkinsonism Relat Disord.

[CR31] Oba H, Yagishita A, Terada H, Barkovich AJ, Kutomi K, Yamauchi T, Furui S, Shimizu T, Uchigata M, Matsumura K, Sonoo M, Sakai M, Takada K, Harasawa A, Takeshita K, Kohtake H, Tanaka H, Suzuki S (2005). New and reliable MRI diagnosis for progressive supranuclear palsy. Neurology.

[CR32] Peterson KA, Patterson K, Rowe JB (2021). Language impairment in progressive supranuclear palsy and corticobasal syndrome. J Neurol.

[CR33] Pillas M, Selai C, Quinn NP, Lees A, Litvan I, Lang A, Bower J, Burn D, Low P, Schrag A (2016). Development and validation of a carers quality-of-life questionnaire for parkinsonism (PQoL Carers). Qual Life Res.

[CR34] Quattrone A, Nicoletti G, Messina D, Fera F, Condino F, Pugliese P, Lanza P, Barone P, Morgante L, Zappia M, Aguglia U, Gallo O (2008). MR imaging index for differentiation of progressive supranuclear palsy from Parkinson disease and the Parkinson variant of multiple system atrophy. Radiology.

[CR35] Ransmayr G, Hermann P, Sallinger K, Benke T, Seiler S, Dal-Bianco P, Marksteiner J, Defrancesco M, Sanin G, Struhal W, Guger M, Vosko M, Hagenauer K, Lehner R, Futschik A, Schmidt R (2018). Caregiving and caregiver burden in dementia home care: results from the prospective dementia registry (PRODEM) of the Austrian Alzheimer society. J Alzheimers Dis.

[CR36] Rebeiz JJ, Kolodny EH, Richardson EP (1968). Corticodentatonigral degeneration with neuronal achromasia. Arch Neurol.

[CR37] Robinson BC (1983). Validation of a caregiver strain index. J Gerontol.

[CR38] Schmid NS, Ehrensperger MM, Berres M, Beck IR, Monsch AU (2014). The extension of the German CERAD neuropsychological assessment battery with tests assessing subcortical, executive and frontal functions improves accuracy in dementia diagnosis. Dement Geriatr Cogn Dis Extra.

[CR39] Schmotz C, Richinger C, Lorenzl S (2017). High burden and depression among late-stage idiopathic Parkinson disease and progressive supranuclear palsy caregivers. J Geriatr Psychiatry Neurol.

[CR40] Schneider J, Murray J, Banerjee S, Mann A (1999). EUROCARE: a cross-national study of co-resident spouse carers for people with Alzheimer’s disease: I-Factors associated with carer burden. Int J Geriatr Psychiatry.

[CR41] Schreiner AS, Morimoto T, Arai Y, Zarit S (2006). Assessing family caregiver’s mental health using a statistically derived cut-off score for the Zarit Burden Interview. Aging Ment Health.

[CR42] Southi N, Honan CA, Hodges JR, Piguet O, Kumfor F (2019). Reduced capacity for empathy in corticobasal syndrome and its impact on carer burden. Int J Geriatr Psychiatry.

[CR43] Steele JC, Richardson JC, Olszewski J (1964). Progressive supranuclear palsy: a heterogeneous degeneration involving the brain stem, Basal Ganglia and cerebellum with vertical gaze and pseudobulbar palsy, nuchal dystonia and dementia. Arch Neurol.

[CR44] Tessitore A, Marano P, Modugno N, Pontieri FE, Tambasco N, Canesi M, Latorre A, Lopiano L, Sensi M, Quatrale R, Solla P, Defazio G, Melzi G, Costanzo AM, Gualberti G, di Luzio PU, Antonini A (2018). Caregiver burden and its related factors in advanced Parkinson’s disease: data from the PREDICT study. J Neurol.

[CR45] Tinetti ME (1986). Performance-oriented assessment of mobility problems in elderly patients. J Am Geriatr Soc.

[CR46] Tsai CF, Hwang WS, Lee JJ, Wang WF, Huang LC, Huang LK, Lee WJ, Sung PS, Liu YC, Hsu CC, Fuh JL (2021). Predictors of caregiver burden in aged caregivers of demented older patients. BMC Geriatr.

[CR47] Uttl B, Santacruz P, Litvan I, Grafman J (1998). Caregiving in progressive supranuclear palsy. Neurology.

[CR48] Whiting S, Lincoln N (1980). An ADL assessment for stroke patients. Br J Occup Ther.

[CR49] Wiblin L, Durcan R, Lee M, Brittain K (2017). The importance of connection to others in QoL in MSA and PSP. Parkinsons Dis.

[CR50] Williams DR, Lees AJ (2009). Progressive supranuclear palsy: clinicopathological concepts and diagnostic challenges. Lancet Neurol.

[CR51] Wynn MJ, Carpenter BD (2020). Frontotemporal dementia knowledge scale: development and preliminary psychometric properties. Alzheimer Dis Assoc Disord.

[CR52] Yesavage JA, Brink TL, Rose TL, Lum O, Huang V, Adey M, Leirer VO (1982). Development and validation of a geriatric depression screening scale: a preliminary report. J Psychiatr Res.

[CR53] Zarit SH, Todd PA, Zarit JM (1986). Subjective burden of husbands and wives as caregivers: a longitudinal study. Gerontologist.

